# Impacts of mobility disability and high and increasing body mass index on health-related quality of life and participation in society: a population-based cohort study from Sweden

**DOI:** 10.1186/1471-2458-14-381

**Published:** 2014-04-17

**Authors:** Marianne Holmgren, Anna Lindgren, Jeroen de Munter, Finn Rasmussen, Gerd Ahlström

**Affiliations:** 1 The Swedish Institute for Health Sciences, Department of Health Sciences, Lund University, P.O. Box 187, SE-221 00 Lund, Sweden; 2 Centre for Mathematical Sciences, Lund University, P.O. Box 118, SE-221 00 Lund, Sweden; 3 Department of Public Health Sciences, Karolinska Institutet, P.O. Widerströmska huset, Tomtebodavägen 18A, plan 9, SE-171 77 Stockholm, Sweden

**Keywords:** Mobility disability, Overweight, Obesity, Prevalence, Population study, Health-related quality of life (HRQoL), Participation

## Abstract

**Background:**

Increasing obesity in adults with mobility disability has become a considerable health problem, similar to the increasing trend of obesity in the general population. The aims of this study were to investigate the association of mobility disability with overweight status and obesity in a large population-based Swedish cohort of adults, and to investigate whether mobility disability, high body mass index (BMI), and increasing BMI over time are predictors of health-related quality of life and participation in society after 8 years of follow-up.

**Methods:**

The study cohort included 13,549 individuals aged 18–64 years who answered questions about mobility disability, weight, height, health-related quality of life and participation in society in the Stockholm Public Health Survey 2002 and 2010. The cohort was randomly selected from the population of Stockholm County, and divided into six subgroups based on data for mobility disability and overweight status. Multiple binary logistic regression analyses were performed to assess the likelihood for low health-related quality of life and lack of participation.

**Results:**

Respondents with mobility disability had a higher mean BMI than those without mobility disability. Respondents both with and without mobility disability increased in BMI, but with no significant difference in the longitudinal changes (mean difference: 0.078; 95% CI: -0.16 - 0.32). Presence of mobility disability increased the risk of low health-related quality of life and lack of participation in 2010, irrespective of low health-related quality of life and lack of participation in 2002. The risk of pain and low general health (parts of health-related quality of life) increased for every 5 units of higher BMI reported in 2010. In respondents without low general health at baseline, the risk of obtaining low general health increased for every 5 units of higher BMI in 2010 (OR:1.60; CI: 1.47 - 1.74).

**Conclusions:**

The greatest risk of low general health after 8 years was observed for respondents with both mobility disability and high BMI. These results indicate the importance of working preventively with persons with mobility disability and overweight status or obesity based on the risk of further weight gain.

## Background

Several studies worldwide have reported a high prevalence of obesity among individuals with mobility disability [1-4]. Disability is an umbrella term for impairments, activity limitations, and participation restrictions, reflecting a complex interplay between a person’s impairments of bodily functions and features of the society in which they live [5]. According to the World Health Organization (WHO) Health Survey, about 16% of the world’s population aged 18 years or older is estimated to live with some form of disability. In the United States, the corresponding figure is nearly 22%, and 10% of the population has a mobility disability [6]. The prevalence of any type of disability in the Swedish adult general population aged 16–84 years is about 23%, and 8% have a mobility disability [3]. Prevalence estimates of disabilities vary across countries owing to differences in definitions, inclusion criteria, assessment methods, differences in health services and habilitation, and differences in occurrence of underlying impairments. Therefore, comparisons between specific national data and the results from the WHO Health Survey should be performed with caution [5].

The prevalence of obesity in the worldwide adult population has been estimated at around 10% [7], which is close to recent Swedish estimates [8]. Increasing obesity in adult people with mobility disability has become a considerable health problem, similar to the increasing trend of obesity in the general population [1]. Overweight status and obesity in adults increase the risk for arthrosis in the hips and legs and consequently pain, and also the risk for cardiovascular diseases and diabetes, which may lead to an earlier death [9]. The WHO defines normal weight as a body mass index (BMI) range of 18.5 to 24.9 kg/m^2^, overweight status as a BMI range of 25.0 to 29.9 kg/m^2^, and obesity as a BMI of ≥30 kg/m^2^[5].

Previous studies have reported that obesity and disability defined as the presence of any limitation of activities and/or need for assistive equipment [10] are separately associated with impaired overall health-related quality of life (HRQoL) [11-13]. HRQoL comprises aspects of experienced quality of life that can be related to illness and disease, either physical or mental [14]. Furthermore, the consequences of mobility disability combined with obesity are largely unexplored in terms of participation in society, despite comprehensive research results related to mobility disability per se. Participation in different aspects of social life, such as working life, is a key issue in disability research [15]. Based on published literature, the hypothesis in this study was that overweight status, obesity, and increasing BMI over time increase the risk for lower HRQoL and lack of participation in society to higher degrees in individuals with mobility disability than those without this functional limitation. Therefore, the aims of the present study were twofold: 1) to investigate mobility disability, overweight status, and obesity in a large population-based Swedish cohort of adults, and 2) to investigate whether mobility disability, high BMI, and increasing BMI over time are predictors of low HRQoL and lack of participation in society after 8 years of follow-up.

## Methods

### Design

This study was a population-based longitudinal cohort study in Sweden designed to follow the development of HRQoL and participation in society in 2002 and 2010. The research was based on the Stockholm Public Health Survey. The data collection was managed by Statistic Sweden on behalf of Stockholm County Council and in collaboration with researchers based at the Department of Public Health Sciences, Karolinska Institutet. The cohort is a resource for epidemiological research and available for specific studies after approval from the Stockholm Regional Ethical Review Board and the Stockholm Public Health Cohort Steering Committee. Both the steering committee and the Regional Ethics Committee, Stockholm, Sweden (Dnr: 2012/1193-31/5) approved this study.

### Study populations

The cohort was based on the Stockholm Public Health Survey 2002 and followed up in 2010. The sample was selected by stratified random sampling on sex and residence area. The cohort comprised individuals aged 18–84 years at baseline who were registered in the County of Stockholm. The Stockholm population comprised 1 850 467 inhabitants in 2002 [16]. The total size of the sample was 49,909 individuals in 2002, of whom 31,182 individuals participated in the survey [17]. In the follow-up questionnaire in 2010, 19,128 individuals responded, aged 26–92 years.

**Figure 1:**
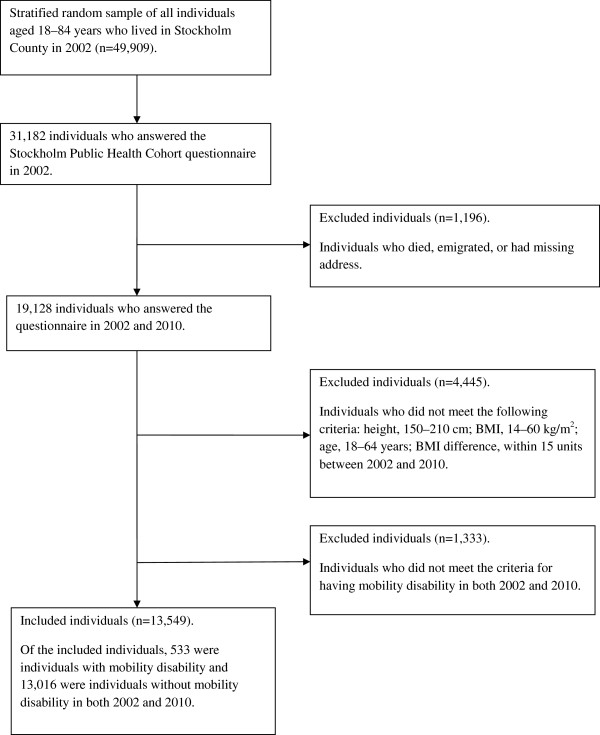
Flowchart for participating individuals in the Stockholm Public Health Survey 2002 and 2010.

In this study, the inclusion criteria were age range of 18–64 years in 2002, BMI range of 14–60 kg/m^2^, height range of 150–210 cm, and complete data in 2002 for the EQ-5D scale, a well-established worldwide short measure of HRQoL [18]. The participants with extreme values for height (less than 150 cm or greater than 210 cm, n = 175), or BMI (less than 14 kg/m^2^ or greater than 60 kg/m^2^, n = 23) or extreme change in BMI (change >15 BMI-units between 2002 and 2010, n = 27) were excluded to minimize misclassification. Individuals, who reported mobility disability in only the 2002 or 2010 surveys, were also excluded. Application of these inclusion criteria selected 13,549 individuals of the 19,128 eligible individuals (Figure 1). Mobility disability was defined by the respondents stating “I have some problems in walking about” (moderate) or “I am confined to bed” (extreme) for the mobility question in the EQ-5D in both 2002 and 2010. For the descriptive analyses, the individuals were divided into six subgroups as follows: mobility disability and underweight/normal weight (MDNW); mobility disability and overweight (MDOW); mobility disability and obesity (MDOB); no mobility disability and underweight/normal weight (NMDNW); no mobility disability and overweight (NMDOW); and no mobility disability and obesity (NMDOB). The reason to merge data on underweight and normal weight was because of data scarcity (underweight n = 224 whereof 14 participants with mobility disability).

### Measurements for HRQoL and participation

The Stockholm Public Health survey in 2002 and the follow-up survey in 2010 were conducted as self-administered postal questionnaires, and included questions about HRQoL and participation in society. Self-reported data on weight and height were collected in both 2002 and 2010.

#### Questions for HRQoL

The 12-item General Health Questionnaire (GHQ-12) is a widely used self-reported instrument for detection of mental disorders in the community and non-psychiatric clinical settings, and has been used in several previous health surveys [19,20]. GHQ-12 is a shorter version of GHQ-28, with comparable validity to the longer version, and has repeatedly been used as a screening instrument in population-based research [21]. Each person self-rates from “less than usual” to “much more than usual” on questions for recent experiences of a symptom or behavior [22]. The answers are scored as 0 or 1 point per question [19], providing a maximum of 12 points. A cut-off score of 3 or less is generally considered to reflect “good” mental health [20,22].

One question about general health from the SF-36 instrument was included in the Stockholm Public Health Survey in 2002 and 2010, namely “How would you rate your general health”. The answer alternatives were as follows [23]: 1 = Excellent; 2 = Very good; 3 = Good; 4 = Poor; 5 = Very poor.

Self-rated pain is commonly included in HRQoL. The dichotomous pain measure in the Public Health Survey was based on three questions about pain. The first question was about pain in the upper back and neck. The second question was about pain in the lower back and the third question was about pain in the shoulders and arms. If the respondents reported pain for more than a couple of days a week in at least one of the three questions, the respondents were considered to have pain.

#### Questions about participation

In the present study, participation was defined as taking part at the labor market and/or in society. The two questions that formed the base for participation were “What is your main employment right now?” and “In the past 12 months, have you more or less regularly participated in activities in society together with several other people?” The dichotomous answer alternatives were “Yes” or “No” [20].

### Statistical analysis

The descriptive analyses were based on the six subgroups set up in 2002. Differences in frequencies between the groups were analyzed using the chi-square-test. Changes in BMI within the groups were tested using a paired *t*-test. An independent-sample *t*-test, assuming unequal variances, was conducted to compare the BMI increases between two groups, and to determine whether the group with mobility disability showed greater increases than the group without mobility disability. Multiple binary logistic regression analyses were performed to assess the impacts of specific factors (sex, age, mobility disability, BMI in 2010, and change in BMI from 2002 to 2010) on the likelihood of the respondents reporting that they had each of the outcome variables (pain, low general health, low mental health, and lack of participation). The SF-36 question about general health with five alternative answers was dichotomized into good and bad health. Good health included the alternatives of excellent, very good, and good, and bad health included the alternatives of poor and very poor. For the regression analyses, dichotomous variables were created for mobility disability (0 = No; 1 = Yes), pain (0 = No pain; 1 = Pain), general health and mental health (0 = Good; 1 = Bad/Low), and lack of participation (0 = No; 1 = Yes). The regression analyses always retained sex and age, while using stepwise elimination of mobility disability, BMI in 2010, and change in BMI from 2002 to 2010, as well as all interaction terms between mobility disability, BMI in 2010, and change in BMI from 2002 to 2010. The interaction terms were not significant in any of the models, and were thus dropped by the backward elimination. In this study there were a rather large number of potential covariates and therefore the order of the eliminations was determined using the Bayesian Information Criterion penalizing more complex models. Statistical analyses were performed using SPSS Version 21.0 for Windows as well as R version 2.15.2 [24].

## Results

### Prevalences of mobility disability and weight statuses

The descriptive analyses showed an unequal distribution between the groups with and without mobility disability with regard to weight in 2002. The respondents with mobility disability had higher prevalences of overweight status and obesity, with the largest disparity in obesity (Table 1).

**Table 1:** Distribution of overweight status and obesity in people with and without mobility disability in 2002 (n = 13,549)

	**With mobility disability**	**Without mobility disability**	**χ**^**2 **^**test**
	**% (n = 533)**	**% (n = 13,016)**	
Normal weight	37.0 (197)	62.7 (8162)	
Overweight	37.9 (202)	30.8 (4006)	
Obese	25.1 (134)	6.5 (848)	P < 0.001

The characteristics of the six subgroups in 2002 are shown in Table 2. There was a large overall proportion of respondents with university education (45%), but a significantly lower number in the groups with mobility disability than in the groups without mobility disability (p < 0.001). In addition, the respondents with mobility disability took part in the labor market to a lower extent than the respondents without mobility disability (p < 0.001). The respondents with mobility disability showed a significantly higher rate of foreign-born respondents (p < 0.001).

**Table 2:** Population characteristics in 2002

	**All**	**MDNW**	**MDOW**	**MDOB**	**NMDNW**	**NMDOW**	**NMDOB**	**p-value**
	**% (n = 13,549)**	**% (n = 197)**	**% (n = 202)**	**% (n = 134)**	**% (n = 8,162)**	**% (n = 4,006)**	**% (n = 848)**	
**Sex, % (n)**								
Female	56.9 (7716)	67.5 (133)	52 (105)	61.2 (82)	64.8 (5291)	41.6 (1665)	51.9 (440)	<0.001
Men	43.1 (5833)	32.5 (64)	48 (97)	38.8 (52)	35.2 (2871)	58.4 (2341)	48.1 (408)	
**Age, % (n)**								
18–36 years	32.5 (4405)	14.7 (29)	4.5 (9)	3.0 (4)	39.7 (3243)	22.9 (918)	23.8 (202)	<0.001
37–56 years	48.2 (6526)	51.8 (102)	49.5 (100)	46.3 (62)	45.4 (3703)	52.3 (2096)	54.6 (463)	
57–64 years	19.3 (2618)	33.5 (66)	46.0 (93)	50.7 (68)	14.9 (1216)	24.8 (992)	21.6 (183)	
**Education, % (n)**								
Primary education	10.9 (1477)	21.8 (43)	26.7 (54)	27.6 (37)	7.9 (644)	14.1 (566)	15.7 (133)	<0.001
Low secondary education	23.3 (3157)	28.4 (56)	34.7 (70)	38.1 (51)	19.7 (1606)	27.6 (1104)	31.8 (270)	
High secondary education/College	19.9 (2702)	15.2 (30)	12.9 (26)	12.7 (17)	21.0 (1715)	18.9 (756)	18.6 (158)	
University	44.8 (6075)	30.5 (60)	21.8 (44)	19.4 (26)	50.5 (4123)	38.5 (1542)	33.0 (280)	
Other	0.4 (49)	2.5 (5)	1.0 (2)	0.7 (1)	0.4 (29)	0.2 (10)	0.2 (2)	
No answer	0.7 (89)	1.5 (3)	3.0 (6)	1.5 (2)	0.6 (45)	0.7 (28)	0.6 (5)	
**Employment status, % (n)**								
In work	81.6 (11058)	43.7 (86)	46.5 (94)	37.3 (50)	81.9 (6682)	86.0 (3444)	82.8 (702)	<0.001
Retired	4.5 (612)	36.0 (71)	40.1 (81)	41.0 (55)	2.4 (195)	4.2 (170)	4.7 (40)	
Student	6.7 (902)	3.6 (7)	1.5 (3)	1.5 (2)	9.0 (731)	3.2 (130)	3.4 (29)	
Unemployed	2.6 (357)	5.1 (10)	2.5 (5)	3.7 (5)	2.6 (211)	2.3 (92)	4.0 (34)	
Other	2.9 (392)	8.6 (17)	7.9 (16)	11.2 (15)	2.6 (214)	2.7 (108)	2.6 (22)	
No answer	1.7 (228)	3.0 (6)	1.5 (3)	5.2 (7)	1.6 (129)	1.5 (62)	2.5 (21)	
**Country of birth, % (n)**								
Born in Sweden	86.3 (11699)	68.5 (1359)	68.8 (139)	67.2 (90)	87.9 (7171)	86.1 (3449)	84.3 (715)	<0.001
Born abroad	13.2 (1794)	31.0 (61)	30.2 (61)	32.1 (43)	11.7 (952)	13.7 (549)	15.1 (128)	
No answer	0.49 (56)	0.5 (1)	1.0 (2)	0.7 (1)	0.5 (39)	0.2 (8)	0.6 (5)	

MDNW: mobility disability and underweight/normal weight. MDOW: mobility disability and overweight. MDOB: mobility disability and obesity. NMDNW: no mobility disability and underweight/normal weight. NMDOW: no mobility disability and overweight. NMDOB: no mobility disability and obesity.

### HRQoL and participation in society in 2002

As shown in Table 3, the respondents with mobility disability (MDNW, MDOW, and MDOB) had significantly lower self-rated HRQoL and participation in society than the respondents without mobility disability (NMDNW, NMDOW, and NMDOB) in 2002 (p < 0.001). In addition, there were substantial differences between the three groups of NMDNW, NMDOW, and NMDOB with regard to low general health in SF-36, meaning that overweight status and obesity differed more between the weight groups than for the respondents with mobility disability.

**Table 3:** Perceived HRQoL and lack of participation in society among the six subgroups in 2002 (n = 13,549)

	**MDNW (n = 197)**	**MDOW (n = 202)**	**MDOB (n = 134)**	**NMDNW (n = 8,162)**	**NMDOW (n = 4,006)**	**NMDOB (n = 848)**	**p-value**
	**% (n)**	**% (n)**	**% (n)**	**% (n)**	**% (n)**	**% (n)**	
**HRQoL**							
Pain (more than a couple of days a week)	70.6 (139)	75.2 (152)	70.9 (95)	24.9 (2034)	27.0 (1082)	30.3 (257)	<0.001
Low general health, SF-36	82.0 (159)	84.6 (170)	85.0 (113)	15.7 (1268)	20.9 (827)	32.7 (276)	<0.001
Low mental health, GHQ-12	42.2 (81)	39.6 (78)	38.9 (51)	23.5 (1902)	18.8 (745)	22.3 (188)	<0.001
**Participation**							
No participation in society	27.9 (55)	28.7 (58)	32.8 (44)	3.5 (284)	4.1 (165)	7.1(60)	<0.001

MDNW: mobility disability and underweight/normal weight. MDOW: mobility disability and overweight. MDOB: mobility disability and obesity. NMDNW: no mobility disability and underweight/normal weight. NMDOW: no mobility disability and overweight. NMDOB: no mobility disability and obesity.

### Increases in BMI

The mean BMI for the whole study population (n = 13,549) was 24.49 kg/m^2^ (SD: 3.67) in 2002 and 25.20 kg/m^2^ (SD: 3.94) in 2010. In 2002, the mean BMI was 27.08 kg/m^2^ (SD: 5.37) for the respondents with mobility disability and 24.38 kg/m^2^ (SD: 3.54) for the respondents without mobility disability. In 2010, the mean BMI was 27.72 kg/m^2^ (SD: 5.89) for the respondents with mobility disability and 25.10 kg/m^2^ (SD 3.80) for the respondents without mobility disability. The results showed a significant increase in BMI from 2002 to 2010 in the group with mobility disability (mean: 0.637; 95% CI: 0.402 to 0.873) as well as in the group without mobility disability (mean: 0.715; 95% CI: 0.680, 0.750). Both groups increased in BMI, but there was no significant difference between the longitudinal changes (mean difference: 0.078; 95% CI: -0.16 to 0.32).

### Predictors for obtaining low HRQoL and lack of participation in 2010

The risk of obtaining pain in 2010, after not reporting pain in 2002, was higher in respondents with mobility disability (OR: 2.98), regardless of BMI (Table 4). A higher BMI in 2010 increased the risk with an OR of 1.12 for every 5 units of higher BMI, regardless of mobility disability. In addition, the risk of pain was further increased for middle-aged (OR: 1.13) and female (OR: 1.41) respondents.

**Table 4:** Risk of low HRQoL and lack of participation in society in 2010, but not in 2002

	**HRQoL**			**Participation**
	**Pain**	**Low general health**	**Low mental health**	**Lack of participation**
	**OR (CI)**	**OR (CI)**	**OR (CI)**	**OR (CI)**
Male	1	1	1	1
Female	1.41 **1.26, 1.58**	1.16 **1.02, 1.31**	1.43 **1.26, 1.62**	0.89 0.78, 1.01
Aged 26–44 years in 2010	1	1	1	1
Aged 45–64 years in 2010	1.13 **1.01, 1.28**	1.03 0.90, 1.17	0.57 **0.50, 0.65**	2.64 **2.13, 3.31**
Aged 65+ years in 2010	0.88 0.74, 1.04	0.90 0.74, 1.08	0.24 **0.20, 0.30**	12.00^1^**9.69, 15.01**
No mobility disability	1	1	1	1
Mobility disability	2.98 **2.09, 4.22**	6.19 **3.93, 9.73**	2.77 **2.04, 3.72**	3.88 **3.03, 4.95**
Higher BMI in 2010 (5 units)	1.12 **1.05, 1.20**	1.60 **1.47, 1.74**	-	-
BMI increase (5 units) between 2002 and 2010	-	1.37 **1.17, 1.62**	-	-

^1^Retired respondents.

Significant values are shown in bold text.

The presence of mobility disability increased the risk of low general health in 2010, after not reporting low general health in 2002, with an OR of 6.19, regardless of BMI (Table 4). In addition, the risk increased with an OR of 1.60 for every 5 units of higher BMI in 2010. Furthermore, a BMI increase of 5 units from 2002 to 2010 increased the risk with an OR of 1.37. Consequently, for a respondent with mobility disability, with 5 units of higher BMI, who also increased their BMI by 5 units from 2002 to 2010, the risk of low general health in 2010 would increase with an OR of 13.57 (6.19 × 1.60 × 1.37), compared with a respondent without mobility disability, and 5 units of lower BMI in 2010, or constant BMI between 2002 and 2010. However, the number of respondents with this high risk was very small (n = 10). With regard to low general health, an additional increase was seen for female respondents (OR: 1.16).

The risk of obtaining low mental health in 2010, after not reporting low mental health in 2002, was higher in respondents with mobility disability (OR: 2.77). An increased risk of obtaining low mental health was also seen for female respondents (OR: 1.43). However, the risk for obtaining low mental health, decreased with an OR of 0.57 for respondents aged over 45 years and an OR of 0.24 for respondents aged over 65 years. Neither BMI alone nor increase in BMI was significantly associated with low mental health, and these items were dropped from the regression model (Table 4).

Regarding lack of participation in society, mobility disability increased the risk of lack of participation in 2010, after not reporting lack of participation in 2002, with an OR of 3.88. Neither BMI alone nor increase in BMI was significant. For respondents aged 45–64 years, the risk increased with an OR of 2.64.

### Predictors for retaining low HRQoL and participation in society in 2010

After reporting pain in 2002, mobility disability increased the risk of retaining pain in 2010 (OR: 4.51), regardless of the change in BMI between 2002 and 2010 (Table 5). Five units of higher BMI in 2010 increased the risk with an OR of 1.14. For female and middle-aged respondents, the risk of retaining pain increased with an OR of 1.41 and 1.27, respectively.

**Table 5:** Risk of long-term low HRQoL and lack of participation in society in 2010 when also reported in 2002

	**HRQoL**			**Participation**
	**Pain**	**Low general health**	**Low mental health**	**Lack of participation**
	**OR (CI)**	**OR (CI)**	**OR (CI)**	**OR (CI)**
Male	1	1	1	1
Female	1.41 **1.23, 1.63**	1.03 0.87, 1.21	1.30 **1.10, 1.54**	0.99 0.71, 1.39
Aged 26–44 years in 2010	1	1	1	1
Aged 45–64 years in 2010	1.27 **1.08, 1.49**	1.50 **1.24, 1.82**	0.88 0.75, 1.01	3.35 **2.04, 5.65**
Aged 65+ years in 2010	1.04 0.86, 1.27	1.11 0.89, 1.39	0.40 **0.29, 0.54**	5.49^1^**3.33, 9.32**
No mobility disability	1	1	1	1
Mobility disability	4.51 **3.48, 5.90**	8.45 **6.17, 11.84**	3.00 **2.22, 4.06**	2.97 **1.98, 4.52**
Higher BMI in 2010 (5 units)	1.14 **1.05, 1.24**	1.18 **1.07, 1.31**	-	-
BMI change away from average (5 units/unit) between 2002 and 2010	-	1.23 **1.08, 1.41**	-	-

^1^Retired respondents.

Significant values are shown in bold text.

Regarding general health, mobility disability increased the risk of retaining low general health in 2010, after reporting low general health in 2002, with an OR of 8.45, regardless of BMI (Table 5). Furthermore, the risk increased with an OR of 1.18 for every 5 units of higher BMI in 2010. In addition, an increase to above average BMI or a decrease to below average BMI by 5 units, i.e. away from 25 kg/m^2^, both increased the risk with an OR of 1.23. Besides, the risk of retaining low general health also increased for age of 45–64 years (OR: 1.50).

The risk of retaining low mental health and not participating in society in 2010 increased with an OR of 3.00 and 2.97, respectively, in respondents who reported that they had mobility disability (Table 5). The BMI factor was not significant. An increased risk for female respondents to retain low mental health was seen (OR: 1.30). For respondents who lacked participation in society, the risk of retaining the problem increased with an OR of 3.35 and 5.49 for ages of 45–64 and 65+ years, respectively.

## Discussion

Our hypothesis in this study that overweight status, obesity, and increasing BMI over time increase the risks of lower HRQoL and lack of participation in society with long-term mobility disability was partly confirmed. Our results showed different patterns over time when the respondents already had a health problem or lack of participation, compared with when the respondents obtained the problem in addition to mobility disability and/or high BMI. In this study, overweight status and obesity were higher in respondents with mobility disability than in respondents without mobility disability, consistent with previous findings in the literature [1,2,4]. Although BMI is increasing worldwide among individuals with or without mobility disability, to our knowledge, no previous studies have compared the increasing prevalences over time between these two groups in a cohort study in regard to HRQoL and participation in the society. Although the BMI did not show a greater increase in the respondents with mobility disability compared with the respondents without mobility disability in the present study, the respondents with mobility disability were affected by BMI increases to a greater extent with regard to HRQoL and participation in society.

Owing to the association between overweight status/obesity and low general health, the respondents without mobility disability appeared to be more affected than the respondents with mobility disability. More than twice as many respondents without mobility disability rated their general health as low in the group with obesity (NMDOB) than in the group with normal weight (NMDNW). Among the respondents with mobility disability, the differences were only a few percentage points between the group with obesity (MDOB) and the group with normal weight (MDNW). This is in line with previous studies showing associations between low HRQoL and overweight status/obesity [12]. However, although this should affect all of the respondents in the same way, the impact of mobility disability was presumably so great that the effect of BMI was not noticeable. The groups with mobility disability had a very high percentage of participants with low general health.

Over the 8-year period, the increased risk of lower HRQoL for females is in line with previous research [25]. Furthermore, the risk of low general health was substantially both for respondents with mobility disability as well as for respondents with high BMI. In addition, exposure to both mobility disability and high BMI was associated with extra risks or an increased burden (multiplying the ORs), regardless of low general health at baseline or not. This is a significant knowledge to focus on when tailored health promotion programs. The results were perhaps not unexpected because of the well-known associations between overweight status, obesity, and low general HRQoL [11-13], especially self-rated health [11,25], as well as the well-known moderate associations between physical and mental HRQoL among individuals with mobility disability [10]. A less-affected mental health has been seen among individuals with mobility disability in a previous study [26]. The relatively low association between disability and mental health over time can be explained by acceptance of loss, scaling back of goals, and a series of value changes with emphasis on the subjective meaning of the disability [27].

One unique and important finding in our study was that a large increase in BMI over time increased the risk of obtaining and also retaining low general health. A meta-analysis by Ul-Haq et al. have shown that physical HRQoL was reduced in all BMI categories above normal BMI, but mental health was only reduced in class III obese (≥40 kg/m^2^) adults [28]. In our study, no such association was observed between BMI class III and mental health among respondents with or without mobility disability. One explanation could be low statistical power due to few respondents with obesity class III (1.1%) and, as a result, no association was detected in our sample.

This 8-year follow-up study showed an additional effect on HRQoL (pain and low general health) when the BMI increased among people with both mobility disability and high BMI. Besides the risk of low HRQoL (pain and low general health) with high BMI or increase in BMI over time, overweight status and obesity increase the risk of several serious diseases [9]. It is well-known that obesity, as well as other conditions such as depression, social isolation, pain, and fatigue, are secondary conditions to several disabilities [29-31]. Although these secondary conditions exist among people without mobility disability as well as among people with mobility disability, the frequency is much higher for people with mobility disability [31]. A healthy weight is important for avoiding several serious diseases, especially because of the vulnerability that already exists among individuals with long-term mobility disability with regard to HRQoL.

Among persons with mobility disability, the risk of not participating in society was threefold, but no risk was seen for high BMI or increase in BMI (5 units). Independently of the respondents participating in society in 2002, but not in 2010, or if the respondent did not participate in either of these years, the results showed no impact of high BMI or increasing BMI in neither respondents with nor without mobility disability. It is difficult to know why high BMI or increasing in BMI over time didn´t affect the participation in the society but one reason might be that it is only severe, like BMI class III, obesity that has a substantial effect. As described previously, in our study, there may be too few respondents with BMI class III obesity (1.1%). Independently of high BMI or not, it is well-known that individuals with mobility disability have difficulties participating in society because of diverse obstacles [5], such as environmental barriers [32]. In any case, Crawford et al. [33] found that individuals with mobility disability who reported high levels of physical activity participated in social activities to a higher degree than those who were less physically active. These findings may have public health implications, and individuals with mobility disability could be encouraged to participate more actively in society. Besides the increase in social activity through physical activity, physical activity also improves psychological wellbeing and reduces the risk of preventable health conditions [34].

We found that it is worse to stop participating than not to participate at all. The risk of stopping participation was almost four times higher for the respondents with mobility disability who reported participating in 2002 but had stopped participating in 2010 compared with the respondents with mobility disability who retained a lack of participation in 2010 after reporting a lack of participation in 2002, with a risk of nearly three times. The importance of participation in society for individuals with disability is very well-known in the disability research field, and has been transferred into the ICF classification [15].

Age was found to be the greatest predictor of not participating in society in 2010, irrespective of the presence or absence of mobility disability. The respondents who did not report a lack of participation in 2002, but obtained a lack of participation in 2010 had a greater risk of obtaining a lack of participation than the respondents who reported a lack of participation in 2002 and retained a lack of participation in 2010. Hence, if the respondents did not participate in society except for working, their risk of exclusion increased when they retired. In 2010, there were 2,577 respondents (19%) who had retired, and 1,080 of those respondents did not participate in society in other ways. This finding means that the results must be interpreted with great caution based on the objectives of this study.

From the results of the present study, it is not possible to say whether mobility disability or obesity came first. Instead, the results show the complexity of the interactions between mobility disability and overweight status/obesity. This is verified by previous research showing that the interactions are not unusual in both directions, i.e. mobility disability leading to obesity or obesity leading to mobility disability [1]. In any case, we know that the respondents’ HRQoL will be affected by both high BMI and mobility disability, and that these effects are even more distinct in middle-aged respondents and in females.

### Method discussion

A strength of this study is the longitudinal population-based design, which makes it possible to assess the impacts on outcome variables of longitudinal changes in BMI for individuals with mobility disability. One limitation, however, is the lack of information about the kind of mobility disability in the respondents. To exclude temporary mobility disability, we decided to exclude individuals who reported that they had mobility limitations only in 2002 or only in 2010, meaning that the respondents in the present study had long-term mobility disability compared with those without mobility disability.

Self-reported data on weight and height are known to be less precise than measured data [35]. The degree of underreporting increases with increasing body weight [36], and therefore the prevalence of overweight status can be underestimated [37] and may introduce some degree of misclassification bias. However, it seems reasonable to assume that misreporting at the individual level should be rather constant over time. Therefore, the change in BMI over time may be estimated with reasonably good precision. It is also known that BMI may not be the best measurement for individuals with disability because of their lower lean muscle mass [4]. In addition, in this study the category “normal weight” also incorporates the respondents with underweight since the latter subgroup consisted of very few respondents (n = 224 out of which only 14 had mobility disability). A sensitivity analysis using two separate groups showed that the overall results were not affected by this. Furthermore, with regard to the generalizability, there were 7% more women in the sample before and after our exclusions criteria compared to the general Stockholm county population in 2002 [38]. In the younger age 18–36 years, there were nearly 10% fewer respondents in our sample compared to the original population of Stockholm county in 2002. In the groups 37–56 and 57–64 years, there were about 5% more respondents in each group than in the total Stockholm county population. However, given these relative small differences in distribution of age and sex, our primary interest in this study was mainly to present relative associations between mobility disability, increasing body mass index, HRQoL, and participation in society, which we believe to be generalizable to the target population.

The resulting regression models indicated that a significant interaction term could have mitigated this extra risk or added yet more risk, but it was not significant in this data set from the general population of Stockholm. In the models, we used BMI changes in units of 1 BMI. The results are presented in changes of 5 BMI units because of the width of the BMI categories and to assess reasonable sizes of BMI differences that can be easily related to.

Although the participation in this study did not cover all domains, the participation indicators covered important domains such as work and activities. Our definitions for participating in the society were derived from two questions, i.e. taking part in the labor market and/or participating in activities in society. We were limited to using these two questions on employment and participation in activities to determine participation in society. The generalizability of the results in this study could be considered high due to the population based sample of adult people in a larger county. However, the results about disability are only applicable for long-term mobility disability (respondents who only had mobility disability at one of the two data collection occasions were excluded).

## Conclusions

This longitudinal population-based cohort study shows the interaction between mobility disability and overweight status and obesity. The results verify previous research in terms of higher proportion of overweight status and obesity among the respondents with mobility disability compared with the respondents without mobility disability. The respondents with mobility disability also contained a much higher percentage of participants with low HRQoL. The study contributes new findings about the impact over time, when the greatest risk of low general health after 8 years was observed for respondents with both mobility disability and high BMI. This study indicates the importance of working preventively with persons with mobility disability and overweight status/obesity owing to the risk of further weight gain and low general health. More research is needed to provide knowledge for how to support individuals with mobility disability with a view to either losing weight or preventing weight gain, which may result in better/higher HRQoL and a decreased risk for other serious obesity-related diseases.

## Competing interests

The authors declare they have no competing interests.

## Authors’ contributions

All authors participated in the study design, and interpretation of the results. MH performed literature searches, data analysis and drafted the first version of the manuscript. Statistician AL worked closely and supervised MH in the data analyses. AL made the stepwise regression analysis using the Bayesian Information Criterion models. GA, FR and JdM contributed to the content and critical review of the manuscript. All authors made the final decision to submit for publication. All authors read and approved the final manuscript.

## Pre-publication history

The pre-publication history for this paper can be accessed here:

http://www.biomedcentral.com/1471-2458/14/381/prepub
